# The effect of lidocaine iontophoresis for the treatment of tinnitus: a systematic review

**DOI:** 10.1007/s00405-022-07645-8

**Published:** 2022-09-14

**Authors:** Marcus Bülow, Norman Best, Sebastian Brugger, Steffen Derlien, Dana Loudovici-Krug, Christina Lemhöfer

**Affiliations:** Institute for Physiotherapy, University Hospital Jena, Am Klinikum 1, 07743 Jena, Germany

**Keywords:** Electrotherapy, Direct current, Local anesthesia, Tinnitus, Lidocaine

## Abstract

**Purpose:**

Tinnitus is a common symptom with multiple causes and treatment options. Previous studies have investigated the effect of lidocaine iontophoresis. The aim of this review is to systematically present the effects on tinnitus and to derive possible effects.

**Methods:**

In accordance to the PRISMA statement, the search and analysis were performed. An abstract in German or English and a performed intervention with lidocaine iontophoresis for the treatment of tinnitus, independent of the study design, were considered as inclusion criteria. Due to the heterogeneity of the studies, only a narrative synthesis was performed.

**Results:**

The search yielded 179 studies of which 170 were excluded. Six full-texts and three abstracts were included. In total, 957 patients were treated with lidocaine iontophoresis. The percent improvement in symptoms after lidocaine iontophoresis ranged from 4% to 62%. The qualitative assessment of the studies resulted in an overall “weak” rating for all of them.

**Conclusions:**

Due to the heterogeneity and the limited quality of the studies found, no clear statement can be made about the efficacy. The number of those who benefited from therapy varied widely. In addition, it cannot be ruled out that the effect was merely due to electrical stimulation of the cochlea.

## Introduction

### Tinnitus

The widespread disease tinnitus is generally defined as the subjective perception of a sound in the absence of an external sound source, with between 5% and 15% of the total population suffering from this condition [1]. About 1% of the population shows a considerable limitation in the quality of life due to the ringing in the ears. Additional concomitant diseases, such as sleep disorders, depression or anxiety disorders, can lead to negative effects on almost all aspects of daily life [1].

The causes for the development are numerous. Thus, dysfunctions in the auditory system, acute noise trauma, cardiovascular or neurological origins can lead to the disturbing auditory perceptions [2, 3]. Myofascial overload and cervicogenic dysfunction can also lead to tinnitus [4, 5].

The treatment options for tinnitus, such as the causes, are varied and usually involve an interdisciplinary approach [6]. In the European and American guideline for the diagnosis and treatment of tinnitus, cognitive behavioral therapy, sound therapy, pharmacotherapy, and alternative treatment methods are presented in addition to technical aids, such as hearing aids. However, only cognitive behavioral therapy was strongly recommended [7, 8]. It is important to emphasize that due to the variety of causes for tinnitus, not every treatment approach can be equally effective for every affected person.

Earlier publications also reported the possibility of therapy by lidocaine [9]. The first positive influences of lidocaine on tinnitus were discovered rather accidentally during intravenous application in 1935 [9, 10]. In the following years, several studies were conducted on the efficacy of both intravenous administration and using iontophoresis[9]. The positive results after such therapy varied from 0% to 80%. [11, 12]. From the point of view of physiotherapy and especially electrotherapy, a closer look and evaluation of the therapy with iontophoresis is interesting from today's point of view, because it is an easy to enable therapy option.

### Iontophoresis

Iontophoresis is described as the use of a constant direct current for the transcutaneous application of ionized or undissociated agents [13].

It is important that these active ingredients have an electrical charge or are dissolved in a conductive electrolyte [14]. Based on the principle that like charges repel each other, when the ions come into contact with the electrodes, the negatively charged ions are repelled from the cathode and positively charged ions are repelled from the anode into the body. It is, therefore, helpful to think of iontophoresis as the use of an active electrode to "push" similarly charged ions through the skin [15].

This technique was apparently first described by Veratti in 1747 [16]. Furthermore, the technique of iontophoresis was revived at the beginning of the twentieth century by Leduc who introduced the concept of ion therapy and formulated laws that governed this process [16]. He demonstrated that ionic "drugs" can penetrate the skin and exert local and systemic effects, and that the charge of the particular drug is of critical importance [16].

Lidocaine iontophoresis is performed as follows: the patient lies on the non-affected side of the tinnitus. In the case of bilateral tinnitus, treatment is performed for each side in turn. The lidocaine solution (usually in combination with epinephrine or adrenaline) is instilled into the ear canal as well (lidocaine ions are positively charged), and the cathode is attached to the contralateral arm. Subsequently, with the help of a direct current device, the current is applied at a maximum of 4 mA for a few minutes [17].

### Objectives

The aim of this study is to present the effectiveness of lidocaine Iontophoresis approach and to transfer and re-discuss earlier research and approaches to the present time. In addition, in the recent past, individual studies on the topic of iontophoresis treatment have been listed in reviews on the therapy of tinnitus and their effectiveness has been presented [6, 18, 19]. To date, there has not yet been a systematic review on this topic.

## Materials and methods

### Data source

The study was not registered in advance, as the extension of the work to a systematic review only became apparent in the course of the search. All methods used are in accordance with the PRISMA (Preferred Reporting Items for Systematic Reviews and Meta-analyses) statement [20].

A reviewer (MB) conducted systematic literature searches in five electronic databases. These included MEDLINE, The Cochrane Library, Scopus, PEDro, and the ISI Web of Knowledge. In addition, a review of the International and European Clinical Trails Register was performed to find unpublished studies. In addition, a search of the Conference Proceedings Citation Index for gray literature was performed, as well as a screening of all references of included studies to find further potentially relevant studies.

The following keyword combination was used: (tinnitus OR “ear noises”) AND (“iontoph*”).

### Study selection

All studies published by the search date of January 4th, 2022, that had at least one abstract in German or English were included. Further inclusion criteria were intervention studies, independent of design, that performed iontophoresis with lidocaine or a derivative in patients with tinnitus. The cause of symptomatology was not further restricted. A further follow-up session after the intervention was not required.

Relevant studies were imported into the literature management program Citavi 6.10 (Swiss Academic Software GmbH). After automatic and, subsequently, manual removal of duplicate studies, two reviewers (MB and CL) independently examined the titles and abstracts for relevance and inclusion, and later also the full texts. Case of two abstracts missing full texts, requests for the texts were sent to the authors, but neither answered [21]. The journal replied that unfortunately there was no full text to be found on the abstract in question [22]. The references of the included studies were examined at the end for possible additional work not previously found.

### Data extraction

Two reviewers (CL and SB) extracted all relevant parameters from the included studies. These included sample size (intervention and control group), type of tinnitus (pulsatile/non-pulsatile), implementation of iontophoresis in the intervention group, therapy of the control group, if available, outcome parameters, percentage of positive outcomes.

In the final step, the qualitative evaluation of the included studies was performed using the ”Quality Assessment Tool for quantitative Studies" (Effective Public Health Practice Project) [23]. The assessment was performed independently by two reviewers (MB and CL). The study instrument included ratings on the following six components: selection bias, study design, confounders, blinding, data collection methods, and withdrawals/dropouts. The individual ratings were combined into an overall rating that was divided into "strong", "moderate" or "weak" in accordance with the specifications.

Any issues or disagreements throughout the process were discussed and resolved with a third reviewer (SD).

This was followed by a narrative synthesis of the results, as there was considerable heterogeneity among the included studies in the nature of the investigation and inclusion criteria.

## Results

**Fig. 1:**
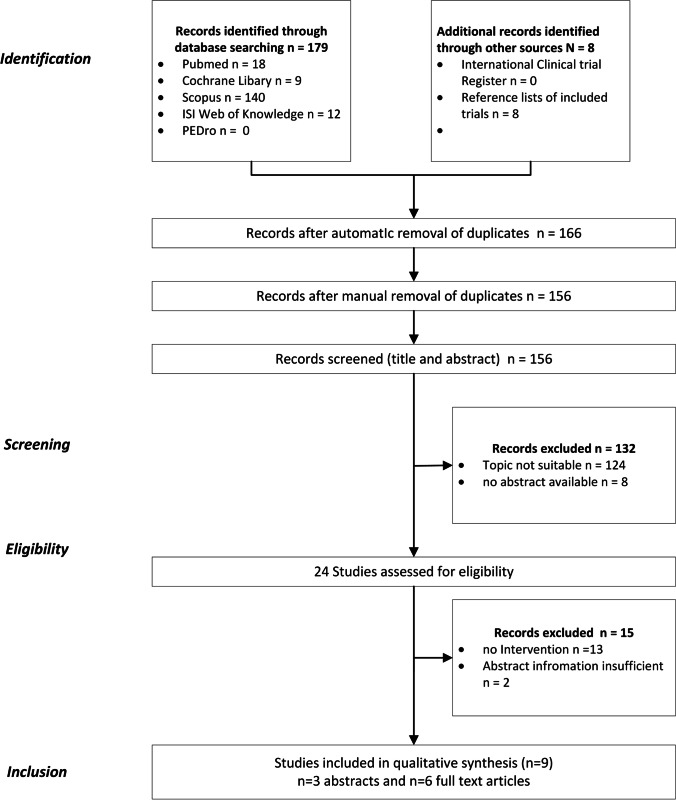
Flow chart showing the study selection process

In total, the search yielded 179 studies. After removal of duplicates, 155 remained. 143 studies did not meet the inclusion criteria and were excluded. The reasons for exclusion are shown in Fig. 1. Two abstracts were excluded after discussion with all involved reviewers, despite the fact that the topic was actually suitable, because the abstract was too short and essential information about the number of patients, the duration of the intervention and the usage of which drug was missing. [24, 25]. The attempt to obtain the missing information or the complete publication by contacting the authors failed. At the end of the process, a total of nine studies were included in further analysis. Six were available as full text [10, 17, 26–30] and 3 more as abstract [31–33]. From the references of included studies, eight potentially suitable studies were identified, but after closer inspection, they did not meet the inclusion criteria and were, therefore, excluded.

### Qualitative assessment

The assessment of the included studies with the Quality Assessment Tool resulted in a "weak" overall rating for the six studies for which a full text was available (Table 1). The studies for which only an abstract was available were not assessed due to missing information.

**Table 1:** Qualitative assessment of includes full-texts "Quality Assessment Tool" (Effective Public Health Practice Project)

	Selection bias	Study design	Confounders	Blinding	Data collection methods	Withdrawals/ dropouts	Global rating
Brusis 1985 [10]	Weak	Moderate	Weak	Weak	Weak	Strong	Weak
Laffree 1998 [26]	Weak	Strong	Weak	Moderate	Weak	Strong	Weak
Schönweiler 1987 [27]	Moderate	Moderate	Weak	Weak	Weak	Weak	Weak
Wedel 1991 [28]	Weak	Moderate	Weak	Weak	Weak	Weak	Weak
Welkoborsky 1988 [17]	Weak	Moderate	Weak	Weak	Weak	Weak	Weak
Zeuner 1989 [29]	Weak	Moderate	Weak	Moderate	Weak	Weak	Weak

### Data synthesis

In the nine included studies, a total of 957 patients were treated with lidocaine iontophoresis. The inclusion criteria of the individual studies included the duration of the tinnitus as well as previously performed frustrated therapy attempts. In some cases, no inclusion criteria were given [10, 31, 32]. Information on the cause of tinnitus was provided by seven studies, but they did not perform subgroup analyses for this purpose [10, 17, 26–29, 33]. Further differentiation into pulsatile and non-pulsatile Tinnitus was also rarely made. One study included only subjects with non-pulsating tinnitus [27], and two studies included both variants [17, 28]. In the other studies, no statement was made in this regard Lidocaine–iontophoresis was performed with 4% lidocaine and epinephrine in a ratio of 1:2000, resulting in a 2% solution. Two studies indicated a duration of therapy of 10 and 12 min, respectively [17, 29]. The intensity of current used was specified between 0.5 and 4.0 mA [17, 27, 29, 34]. A correlation between higher currents and better effects could not be found, and could not be statistically calculated due to missing data. The frequency of therapy varied from weekly to daily. The outcome parameters were mostly a subjective evaluation of the tinnitus and recorded without standardized measurement instruments. Welkoborsky et al. and Zeuner et al. also used audiometry to measure other parameters, such as hearing ability. One side effect of the therapy reported by Zeuner et al. and Willat et al. was dizziness when switching off the current [29, 32]. Brusis et al. described temporary sensory deafness after the intervention and Welkoborsky et al. described irritation of the auditory canal [10, 17].

4% [29] to 62% [10] of patients reported improvement in symptoms after lidocaine iontophoresis. Iontophoresis with NaCl yielded positive results in 0% [32] to 30% [31] of patients.

The extracted data of the included studies are shown in Table 2. The extraction of data from studies that were only available as abstracts was clearly limited. One of the included studies indicated that the study was approved by a local ethics committee [26].

**Table 2:** Data extraction of includes studies

Study	Country	Full text	No. of participants (intervention/control) [targeted group]	Type of tinnitus (pulsatile and non-pulsatile)	Implementation of iontophoresis in the intervention group				Treatment in the control group	Outcome measure	Specifics
					Concentration of lidocaine	Intensity	Duration	Frequency			
Brusis, 1985 [10]	Germany	Yes	50 (50/0) [Adults]	Not stated	2% with Epinephrine	0.5 mA	11 min	Not stated	No control group	Subjective improvement 4 level scale	
Laffree, 1988 [26]	Netherlands	Yes	46 (24/22) [Adults]	Not stated	2% without Epinephrine	0.7–2.0 mA	10 min	5 consecutive days	Same scheme only with NaCl	Subjective improvement 5 level scale	Lidocaine (intravenous) was administered to all patients before the examination
Mosca, 1997 [33]	Italy	No	20 (20/0) [Adults]	Not stated	Not stated	Not stated	Not stated	Not stated	Control by the same group with NaCL	Not stated	
Schönweiler, 1987 [27]	Germany	Yes	52 (52/0) [Adults]	Non-pulsatile	Not stated	Max. 1 mA	Not Stated	5 consecutive days	No control group	Subjective improvement 6 level scale	Lidocaine (intravenous) administration as well as circulation-enhancing medication was given to some patients before the examination
von Wedel, 1991 [28]	Germany	Yes	168 (168/0) [Adults]	Both	Not stated	Not stated	Not stated	Not stated	No control group	Subjective improvement 6 level scale	Several therapies were investigated in parallel (e.g., electrostimulation, biofeedback)
Welkoborsk, 1988 [17]	Germany	Yes	54 (54/0) [Adults]	Both	2% with Epinephrine	0.5 mA	13 min	4–16 sessions on consecutive days	no control group	subjective improvement 4 level scale	
Willat, 1988 [32]	England	No	40 (40/0) [Adults]	Not stated	2% with Epinephrine	Not stated	Not stated	Once every intervention with weekly intervals	Control by the same group with NaCL and placebo	Subjective improvement	Allocation of treatment order was random
Williat, 1988 [32]	England	No	10 (10/0) [Adults]	Not stated	2% with Epinephrine	not Stated	Not stated	Once every intervention with weekly intervals	Control by the same group with NaCL and placebo	Subjective improvement	Allocation of treatment order was random
Zeuner, 1989 [29]	Germany	Yes	50 (50/0) [Adults]	Not stated	2%	0.5–2 mA	10 min	All Therapies 5 times each, in weekly intervals	Control by the same group with NaCL	Subjective improvement	Several therapies were investigated in parallel (electrostimulation, lidocaine and NaCl Ionophoresis) Lidocaine (intravenous) was administered to all patients before the examination

## Discussion

The present systematic review shows that the existing data on this treatment method is insufficient and of poor quality. Therefore, a clear conclusion regarding the efficacy is not possible. Therefore, the results can only give indications that the treatment of tinnitus with lidocaine by means of iontophoresis is practicable and associated with positive effects in all studies. Although the number of patients with a benefit varied greatly and the classification criteria were recorded without standardized measurement instruments. The results of the individual studies vary considerably in some cases, and positive effects are also seen in the placebo controls. However, despite the results, it is possible that lidocaine iontophoresis may help individual patients. Since the causes are diverse, this is not inferable and should be considered in analyses and inclusion criteria in further studies. Therefore, the question remains open whether a physiological effect is really achieved by the administration of lidocaine or if it is merely an effect of electrotherapy. Wedel et al. postulated that the effect was due to the direct current rather than the lidocaine [30]. This view is also confirmed by recent studies. Thus, it has been shown that intra- and extracochlear electrical stimulation can contribute to the suppression of non-pulsatile tinnitus [35–37]. Therefore, the effect of lidocaine does not seem to provide the only possible explanation. However, a recent systematic review did not find an answer as to which stimulation pattern is most effective for suppression [35]. In this analysis, we could not find any correlation between higher current and better effect, which, therefore, requires further investigation. Marinelli et al. postulated that the results could be the basis for the development of an extracochlear implantable device, which would electrically stimulate the cochlea and thus help reduce tinnitus [36]. The results of the rather historical investigations found, thus have some influence on current science as well.

The extent to which lidocaine can diffuse into the ear was shown by Tolsdroff et al. who successfully used the effect of lidocaine iontophoresis to anesthetize the tympanic membrane [38]. In contrast to infiltration anesthesia, where the local anesthetic is injected in close proximity to the nervous structure, surface anesthesia requires the drug to cover a certain distance. This is where iontophoresis can help, as was postulated as early as 1911 [38]. In the case of lidocaine, the positively charged ion travels from the positively charged anode in the auditory canal to the negatively charged cathode on the contralateral arm, penetrating all the necessary layers until it reaches the nerve [38]. The postulated effect on tinnitus is based, on the one hand, on improved blood flow to the inner ear and, on the other hand, on the concept of sensory epilepsy, in which local anesthetics alleviate abnormal hypersensitivity of the central nervous system [6, 9]. In addition, treatment with the sodium channel blocker is also thought to affect other receptors, such as muscarinic ones, which have high lidocaine sensitivity [12].

Further evidence of the efficacy of treatment of tinnitus with lidocaine was provided by Lyttkens et al. in 1979. They found that lidocaine binds to melatonin in the inner ear and postulated a connection of melatonin in the conversion of mechanical to electrical information. Lidocaine could provide assistance in this process and thus have a positive influence on tinnitus [39]. Further research showed that melatonin content is related to noise-induced hearing loss and provides a form of protection [9]. Recent studies also conclude that melatonin can have a positive impact on tinnitus [6, 40]. We are not aware of any other recent studies on the relationship between lidocaine and melatonin. However, the explanatory complex would support the thesis that several mechanisms of action influence the effect of lidocaine ionophoresis.

In a recently published review, Kim et al. demonstrated that intravenous application of lidocaine can also contribute to an improvement of tinnitus [6]. However, the results were also inconsistent and did not allow a conclusive evaluation. An attempt at therapy with intravenously applied lidocaine should, therefore, only be undertaken after exhausting other therapeutic options [6].

It is also important to provide information about possible side effects. In the included studies for this systematic review, little was written about possible side effects. Brusis et al. and Welkoborsky et al. stated that some subjects experienced sensations or irritation of the auditory canal after therapy [10, 17]. Zeuner et al. and Willat et al. described the appearance of dizziness immediately after treatment [29, 32]. In general, lidocaine, especially when administered intravenously, can cause nausea, drowsiness, and also anxiety. Serious side effects described are respiratory inhibition, pulse drop and convulsions [6]. Further studies in this field would also have to address the possible side effects that can occur with iontophoresis and describe them in more detail. Analogous to intravenous use, certain patient groups, e.g., pregnant women, patients with renal and hepatic dysfunction, and those under 18 years of age, should be excluded from these studies [6].

The results also highlight the importance of standardization in reporting study results related to tinnitus. The American Academy of Otolaryngology—Head and Neck Surgery has provided valuable information in this regard for future studies in its Guideline [8].

### Strengths and limitations

The strengths of the present study lie in the systematic conduct of the search and subsequent assessment of the studies found, in accordance with the PRISMA statement. Additional channels such as direct contact with authors and journals were sought to find additional full texts on abstracts found. Thus, the included literature represents the scientific state of the art on this topic.

However, it must be noted that the lack of some full texts significantly limits the synthesis and conclusion, as not enough information was available on the interventions implemented. Due to the heterogeneity in the study design of the included studies, it was not possible to perform a meta-analysis. Furthermore, the existing weak quality of the intervention studies limits the informative value of this systematic review. From today's perspective, the lack of mention of ethical endorsements of the included studies could lead to a limitation in the strength of the review. However, legal and ethical changes have certainly occurred in recent years, and naming or conducting them was not required at that time. Future studies should clearly state ethical consideration.

## Conclusions

This review shows that there may be a positive effect of lidocaine iontophoresis on chronic tinnitus for some patient. However, due to the limited study quality, the evidence is clearly restricted. Further high-quality studies are needed to clarify the evidence and confirm the clinical relevance. In this context, double-blinding and placebo control should be considered urgently in the study design. Collaboration with other professions such as pharmacologists and biochemists can help to improve the basic understanding of this form of therapy.
